# Evaluation of possible biological control of *Fusarium* *concentricum* sp. using plant extracts and antagonistic species of microbes *in vitro*

**DOI:** 10.12688/f1000research.27098.2

**Published:** 2022-04-27

**Authors:** Mohammed Faruk Hasan, Mohammed Asadul Islam, Biswanath Sikdar

**Affiliations:** 1 Department of Genetic Engineering and Biotechnology, University of Rajshahi, Rajshahi, 6205, Bangladesh

**Keywords:** Fusarium sp., Plants extract, Non-pathogenic microbes, Antagonisms, Biocontrol

## Abstract

**Background:** *Fusarium* *concentricum* is one of the most devastating fungi responsible for fruit and vegetable crops rot worldwide. The present study was designed to find an ecofriendly control measure for pathogenic *Fusarium* *concentricum*, using suitable bioagents.

**Methods:** Medicinal plant extracts were evaluated or their antifungal activities against *Fusarium* *concentricum* using the poisoned food and serial dilution methods. Antagonistic potency of some nonpathogenic microbes was also assessed on *Fusarium* *concentricum* using the dual culture method.

**Results:** Highest inhibition of growth of *Fusarium* *concentricum* was observed with 68.1% (0.389 mg per 90 mm Petri plate) of mycelia on *Coccinia grandis* plant leaf extract, in comparison to the control grown with 100.0% (1.22 mg/dish). The tested plants extract showed MIC values rages of 80-150 µg/ml on the isolated fungus. The highest inhibition of radial growth was observed using *Trichoderma viride *on *Fusarium* *concentricum* (46.01% inhibition).

**Conclusions:** The findings of present study would be benevolent for antifungal drug development to control *Fusarium* *concentricum* causing fruit and vegetable rot.

## Introduction

*Fusarium* species are a large genus of hyaline ﬁlamentous mold fungi, responsible for fruit and vegetable crop rot ( Al-Najada & Gherbawy, 2015; Ziedan *et al*., 2018). *Fusarium* species are deeply invasive and can cause hematogenously disseminated infections with high mortality in neutropenic patients ( Dignani & Anaissie, 2004). Numerous species of *Fusarium* contribute to yield loss and reduced quality to varying degrees by infection with some mycotoxins ( O’Donnell *et al*., 2009). They also cause decay of various fruit in storage and postharvest conditions ( Whiteside *et al*., 1988). The fruit rot caused by *Fusarium* incurs enormous yield losses and is often observed in fields and markets ( Baria *et al*., 2015). The application of antagonistic agents and plant extracts in agriculture are becoming a major focus of plant protection research. Different preservatives or fungicide treatments are frequently applied to manage fruits diseases and decay, which is an alarming health concern ( Munhuweyi *et al*., 2020). Since most chemical fungicides are highly toxic to humans and animals and they frequently cause water and soil pollution ( Al-Najada & Gherbawy, 2015). Moreover, continuous and indiscriminate use is leading to the development of fungicide resistant strains of pathogens ( Tafinta *et al*., 2013). There are some previous reports on plants extract based controlling techniques in Fusarium fungi causing diseases for some crops and plants ( Baria *et al*., 2015; Ziedan *et al*., 2018).

In Bangladesh, fruit rot is a destructive disease caused by *Fusarium* species on pre-harvest and postharvest fruits. To the best of our knowledge, there is no previous research on biological control of this pathogenic fungus. Therefore, the main objective of the study was to find an ecofriendly control system of *Fusarium concentricum* to decrease fruit and vegetable rot in Bangladesh.

## Methods

### Collection of *Fusarium concentricum*

A pure culture of *Fusarium* sp. was previously isolated and identified (Accession No. MT856371) from postharvest *Citrus reticulata* fruit rot ( Hasan *et al*., 2020a). The culture was preserved at the Department of Genetic Engineering and Biotechnology, University of Rajshahi (Rajshahi, Bangladesh).

### *In vitro* assessment of antifungal potential of selected plants

For antifungal activities screening, six healthy, mature medicinal plants, *Allium sativum, Zingiber officinale, Coccinia grandis, Brassica juncea, Ocimum tenuiflorum* and *Hibiscus rosa-sinensis* were collected from Mirzapur, Binodpur and Kajla village, Motihar, Rajshahi, Bangladesh. 

Collected plants were washed with water to remove dust from the plants’ surface and dried in room temperature. Plant extract preparation and fractionation was performed according to the method by Kader *et al.* (2018). Different parts of selected plants- bulb of *Allium sativum;* rhizome of *Zingiber officinale;* leaves of *Coccinia grandis, Brassica juncea, Ocimum tenuiflorum;* and flowers of *Hibiscus rosa-sinensis* plants were cut into small species and ground by blender to form fine powder. The dried powder of the plants (100gm of each plant) were rinsed in methanol (500ml) using a conical flask, and were incubated in a shaking incubator with occasional shaking for fourteen days. The liquid contents were pressed through Markin cloth followed by filtration using Whatman no. 1 filter paper. Obtained filtered liquids were dehydrated *in vacuo* to leave a blackish and sticky mass. The extracts were collected in vials and preserved in a refrigerator at 4°C.

The inhibitory effect of different plant extracts was measured by following the poisoned food technique ( Balamurugan, 2014). For this, 20µg of each plant extract was added to 20ml of potato dextrose agar (PDA) to fill a 90mm size Petri plate and mixed well. After solidification, seven day old 6 mm size fungal plug was placed in the center of the Petri plates. The Petri plates were incubated at 35°C for seven days in static condition.

### Determination of minimum inhibitory concentration (MIC) 

The required MIC values of selected plants extracts were measured through the serial dilution technique ( Hussain *et al.,* 2010). The extract of 1 gm of plant-based methanol leaves was dissolved into 1 ml methanol to obtain this same stock solution. Fungal suspension was taken into each test tube and added 50-150µg of plants extracts. The tested tubes were then incubated for 3 days. The progression of the isolated fungus was detected in tubes in which the strength of the substances was just below the inhibition level.

### *In vitro* antagonistic test

For evaluation of antagonistic effects, six non-pathogenic pure microbe cultures, *Escherichia coli* (pathogenic), *Rhizobium phaseoli, Rhizobium leguminosarum, Neofusicoccum mangifera, Trichoderma viride* and *Pestalotiopsis sp.* were used against *Fusarium concentricum*. The pure microbe cultures were kindly provided by Dr. Md. Salah Uddin, Associate Professor and Director, Microbiology Lab., Department of Genetic Engineering and Biotechnology, University of Rajshahi, as the part of collaboration.

To assess the antagonistic effects, the dual culture technique was used, as previously described ( Vethavalli & Sudha, 2012). A mycelial disc of 6 mm diameter was cut from the periphery of both antagonist cultures and the test pathogen and placed on a Petri plate with PDA media. For the control, only the test pathogen was placed in the centre of a Petri plate. The Petri plates were incubated at 35°C in darkness.

### Data analysis

The inhibition percentage of mycelial growth= [(Gc-Gt)/Gc] × 100; Where, Gc = Mycelial growth in terms of colony diameter in control set, Gt= Mycelial growth in terms of colony diameter in treatment set. The inhibition percentages of *Fusarium* species growth were calculated using the following formula: Inhibition percentage (%) =100× (dc– dt)/dc; Where, dc = radial growth of pathogen in control, dt = radial growth of pathogen in dual culture. Mean values were compared through least significant different test using SAS software, version 9.4M5 (SAS Inc., Cary, NC, USA). All the experiment and test were replicated thrice.

## Results

### *In vitro* screening of extracts presenting antifungal activity

**Figure 1.**
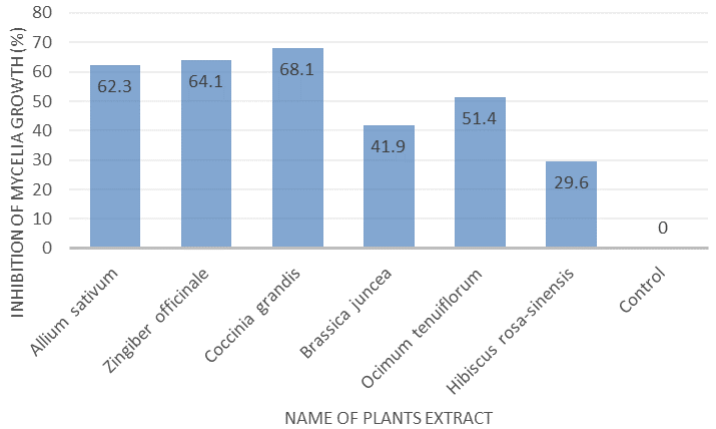
Effect of different methanol plant extracts on percentage inhibition of mycelial growth of *Fusarium concentricum*. Mycelia were collected after seven days of incubation on potato dextrose agar at 35°C in darkness. 90 mm Petri plates were used to culture the tested fungus.

**Figure 2.**
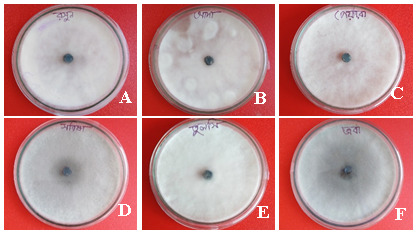
Petri dish images showing the effect of plant extracts on inhibition of mycelial growth of *Fusarium concentricum*. ( **A**) *Allium sativum*, ( **B**) *Zingiber officinale*, ( **C**) *Coccinia grandis,* ( **D**) *Brassica juncea*, ( **E**) *Ocimum tenuiflorum*, and ( **F**) *Hibiscus rosa-sinensis.* Mycelia were collected after seven days of incubation on potato dextrose agar at 35°C in darkness.

All plant extracts showed a degree of growth inhibition of the tested fungus at the same concentrations. The highest inhibition of growth of the isolates was observed at 68.1% of mycelium on *Coccinia grandis*, which was followed by 64.1% on *Allium sativum*, in comparison to the control culture (100.0%). *Hibiscus rosa-sinensis* showed the lowest inhibition of mycelium with 29.6% against the fungal isolate in comparison to the control. The results are presented in Figure 1 and Figure 2.

### Determination of minimum inhibitory concentration (MIC)

**Figure 3.**
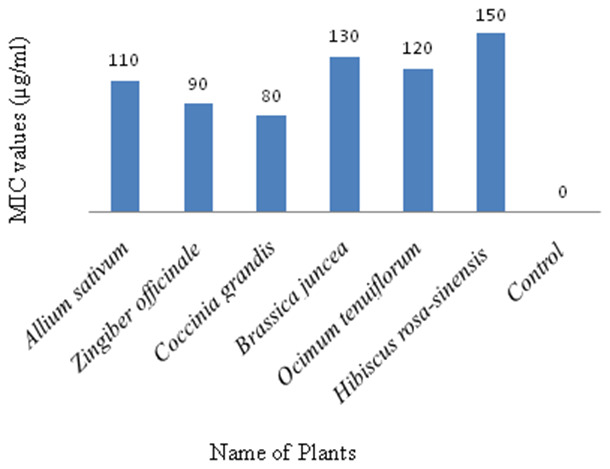
Minimum inhibitory concentration (MIC) of methanol extract showing the effect of plant extracts on inhibition of mycelial growth of *Fusarium concentricum*. ( **A**) Allium sativum, ( **B**) Zingiber officinale, ( **C**) Coccinia grandis, ( **D**) Brassica juncea, ( **E**) Ocimum tenuiflorum, and ( **F**) Hibiscus rosa-sinensis. Mycelia were collected after seven days of incubation on potato dextrose at 35°C in darkness.

The tested plants extracts showed the MIC values ranges from 80-150µg/ml against the fungus *Fusarium concentricum* growth. Highest MIC value of isolated fungus was 80µg/ml on **Coccinia grandis** plant extract and lowest MIC value of isolated fungus was 150 µg/ml on *Hibiscus rosa-sinensis* plant extract ( Figure 3).

### *In vitro* antagonistic assay

**Figure 4.**
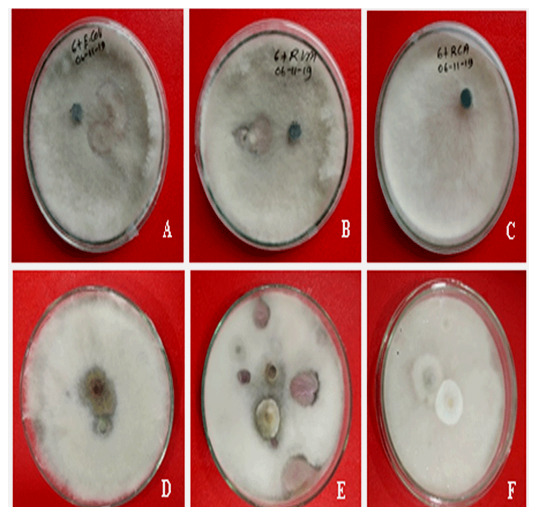
Growth inhibition effects of microbial agents on *Fusarium concentricum*. ( **A**) *Escherichia coli*, ( **B**) *Rhizobium phaseoli,* ( **C**) *Rhizobium leguminosarum*, ( **D**) *Neofusicoccum mangifera,* and ( **E**) *Trichoderma viride* and ( **F**) *Pestalotiopsis sp.*, Mycelia were collected after seven days of incubation on potato dextrose agar at 35°C in darkness.

The highest percentage inhibition of radial growth was observed with *Trichoderma viride* (46.01%) against *Fusarium concentricum* which was followed by 43.33% and 32.05% on *Escherichia coli* and *Rhizobium phaseoli*, respectively ( Figure 4). The antagonistic agent *Rhizobium leguminosarum* did not show any inhibitory activity against the isolated fungus ( Figure 4). The control group also did not show any inhibition of radial growth of *Fusarium concentricum*.

## Discussion

Fruit rot caused by *Fusarium* species is very common in Bangladesh. The main objective of the present study was to study biological control measures for this fungus. Plant extracts are now a superior choice to control different plant pathogens, as reported by several previous studies ( Hasan *et al*., 2020b; Kareem & Al-Araji, 2017; Parveen *et al*., 2014). In our study, we found that the plant extracts *Allium sativum, Zingiber officinale,* and *Coccinia grandis* have significant inhibitory effects on mycelial growth of *Fusarium concentricum*. Hosen & Shamsi (2019) also found significant antifungal activity using *Allium sativum* (53.85%), *Ocimum sanctum* (48.72%) and *Zingiber officinale* (49.35%) against *Fusarium solani* and *F. oxysporum*. Our current results were also supported by data from Khatun *et al.* (2020) and Kareem & Al-Araji (2017). In our present study, *Coccinia grandis* plant leave extract showed a promising MIC capability to control *Fusarium concentricum* fungus. *Piper betle* plant extracts showed a significant antifungal potency on *Fusarium oxysporum* ( Gnanasekaran *et al.*, 2015). For the confirmation of antagonist effects on the radial growth of the pathogen in dual culture have been previously reported by Akhtar *et al.* (2010). By contrast, Nasrin *et al.* (2018) reported 87% inhibition potency on mycelium growth of *Fusarium oxysporum* f. sp. *lycopersici* by *Calotropis proceraon* plant extract. In our study, *Trichoderma viride*, *Escherichia coli*, *Rhizobium phaseoli* and *Alternaria sp.* showed significant antagonistic activity against *Fusarium concentricum*. *Trichoderma viride* showed 45.88% growth inhibition on *Fusarium merismoides* fungi in a study by Hosen & Shamsi (2019), which support our present findings. Nasrin *et al*. (2018) also reported 82% inhibition radial growth by *Trichoderma sp.* against *Fusarium oxysporum* f. sp. *lycopersici.* In contrast, Bashar & Chakma (2014) reported that volatile substances produced by *T. viride*, *A. niger*, *A. flavus* and *A. fumigatus* showed 29.75, 20.15, 15.78 and 12.25% growth inhibition, respectively, on *F. oxysporum*.

In the current investigation, there are some limitations. Although this study showed that some plant extracts and nonpathogenic microbes could control the fungal stain, the number of plant extracts and microbes was limited. In addition, we used only methanol solvent for extraction and did not use other extractions. Moreover, we performed only *in vitro* techniques for antifungal potency screening and did not use any *in vivo* techniques. Therefore, we need to perform further studies to detect ecofriendly control this devastating fungal stain in the future.

## Conclusions

We evaluated different biological control measures for the devastating *Fusarium* fungi. Various medicinal plants extracts and non-pathogenic microbes showed promising inhibitory activities on *Fusarium concentricum in vitro*. These identified control measures of *Fusarium concentricum* shows the importance of further research on *Fusarium* taxonomy to decline the risk of *Fusarium concentricum* caused fruit rot in Bangladesh.

## Data availability

### Underlying data

Figshare: Effect of plant extracts on inhibition of mycelial growth of the *Fusarium* species. https://doi.org/10.6084/m9.figshare.13096262 ( Hasan, 2020a). This project contains the images of Petri plates for each treatment condition.

Figshare: Effect of antagonistic agents on inhibition of mycelial growth of the *Fusarium* species. https://doi.org/10.6084/m9.figshare.13096325 ( Hasan, 2020b). This project contains the images of Petri plates for each treatment condition.

Figshare: Effects of different plants extract by methanol on inhibition percentages of mycelial growth of the Fusarium species, https://doi.org/10.6084/m9.figshare.13134953 ( Hasan, 2020c).

Figshare: Effect of antagonistic agents on inhibition of mycelial growth of the Fusarium species, https://doi.org/10.6084/m9.figshare.13134962 ( Hasan, 2020d).

Data are available under the terms of the Creative Commons Attribution 4.0 International license (CC-BY 4.0).
