# Supplementary material for: Evaluation of possible biological control of Fusarium  concentricum sp. using plant extracts and antagonistic species of microbes in vitro
Source: F1000Res. 2022 Apr 27;9:1394. Originally published 2020 Dec 3. [Version 2] doi: 10.12688/f1000research.27098.2 (PMC9106991; doi:10.12688/f1000research.27098.2)
Supplement: Peer review history

# Peer review: Reviewer response for version 2

**DOI:** 10.5256/f1000research.132272.r136047

**Published:** 2022-05-13

**Authors:** Natarajan Amaresan

**Affiliations:** 1 C. G. Bhakta Institute of Biotechnology, Uka Tarsadia University, Surat, Gujarat, India

Is the work clearly and accurately presented and does it cite the current literature?

Yes

If applicable, is the statistical analysis and its interpretation appropriate?

Yes

Are all the source data underlying the results available to ensure full reproducibility?

Yes

Is the study design appropriate and is the work technically sound?

Partly

Are the conclusions drawn adequately supported by the results?

Yes

Are sufficient details of methods and analysis provided to allow replication by others?

Partly

Reviewer Expertise:

Microbial Diversity, Plant-Microbe interaction, Phytoremediation

I confirm that I have read this submission and believe that I have an appropriate level of expertise to confirm that it is of an acceptable scientific standard.

# Peer review: Reviewer response for version 1

**DOI:** 10.5256/f1000research.29932.r96967

**Published:** 2021-12-15

**Authors:** Mustafa M. El-Zayat

**Affiliations:** 1 Unit of Genetic Engineering and Biotechnology, Mansoura University, Mansoura, Egypt

This research dealt with an important topic in the safe control of the fungus *Fusarium* sp. that causes damage to many types of fruits without the use of chemicals that represent a danger to the environment and health.

- The author should indicate which species of *Fusarium* has been used.
- It is too difficult to extract 100 grams of the plant using only 500 ml methanol.
- It has been mentioned that *E. coli* is not pathogenic while it is pathogenic strain.
- The photos need to be somehow more clear because its resolution is low.

Is the work clearly and accurately presented and does it cite the current literature?

Yes

If applicable, is the statistical analysis and its interpretation appropriate?

No

Are all the source data underlying the results available to ensure full reproducibility?

Partly

Is the study design appropriate and is the work technically sound?

Yes

Are the conclusions drawn adequately supported by the results?

Yes

Are sufficient details of methods and analysis provided to allow replication by others?

Partly

Reviewer Expertise:

Microbiology, Ecology, Nanotechnology, Phytochemistry, Fermentation technology

I confirm that I have read this submission and believe that I have an appropriate level of expertise to confirm that it is of an acceptable scientific standard, however I have significant reservations, as outlined above.

# Peer review: Reviewer response for version 1

**DOI:** 10.5256/f1000research.29932.r89306

**Published:** 2021-07-26

**Authors:** Shaikh Jamal Uddin

**Affiliations:** 1 Pharmacy Discipline, Life Science School, Khulna University, Khulna, Bangladesh

Authors presented the antifungal activity of some plant extract as a eco-friendly control measure for pathogenic *Fusarium* species using the poisoned food method. They found some plant extracts showed promising (>65%) inhibitory activity against *Fusarium* sp. and suggested to use as bioagents to control fungal affected fruit and vegetable rot.

 However, the authors can improve the article if they consider and revise the below points: 

1. Authors did not mention any MIC values. It will be good if they included MIC values of each extracted. 
2. In introduction section author might include a table on the selected plant with their traditional uses and list of active constituents.
3. In the discussion section, there is a lacking of discussion between antifungal activity of selected plant extracts and their traditional uses and reported phytoconstituents. 

Is the work clearly and accurately presented and does it cite the current literature?

Yes

If applicable, is the statistical analysis and its interpretation appropriate?

Yes

Are all the source data underlying the results available to ensure full reproducibility?

Yes

Is the study design appropriate and is the work technically sound?

Yes

Are the conclusions drawn adequately supported by the results?

Yes

Are sufficient details of methods and analysis provided to allow replication by others?

Yes

Reviewer Expertise:

Pharmacological evaluation of ethnomedicinal plants, natural products chemistry and drug discovery

I confirm that I have read this submission and believe that I have an appropriate level of expertise to confirm that it is of an acceptable scientific standard, however I have significant reservations, as outlined above.

# Peer review: Reviewer response for version 1

**DOI:** 10.5256/f1000research.29932.r80330

**Published:** 2021-04-13

**Authors:** Natarajan Amaresan

**Affiliations:** 1 C. G. Bhakta Institute of Biotechnology, Uka Tarsadia University, Surat, Gujarat, India

The authors are reporting some interesting results which will be of significance to the readership however, the manuscript lacks indepth research.

 The effect of the bacteria and botanical extracts should be tested in the pot studies for their effectiveness. *In vitro* studies alone will not warrant their suitability to be used as bioinoculants for management of pathogens.

 Without the *in vivo* studies the work appears as shallow research. The authors may be encouraged to do pot studies and update the manuscript.

Is the work clearly and accurately presented and does it cite the current literature?

Yes

If applicable, is the statistical analysis and its interpretation appropriate?

Yes

Are all the source data underlying the results available to ensure full reproducibility?

Yes

Is the study design appropriate and is the work technically sound?

Partly

Are the conclusions drawn adequately supported by the results?

Yes

Are sufficient details of methods and analysis provided to allow replication by others?

Partly

Reviewer Expertise:

Microbial Diversity, Plant-Microbe interaction, Phytoremediation

I confirm that I have read this submission and believe that I have an appropriate level of expertise to confirm that it is of an acceptable scientific standard, however I have significant reservations, as outlined above.
